# Effect of laser surface remelting on the microstructure and properties of Al-Al_2_Cu-Si ternary eutectic alloy

**DOI:** 10.1038/s41598-017-13953-5

**Published:** 2017-10-18

**Authors:** Bhupendera Prashanth Ramakrishnan, Qian Lei, Amit Misra, Jyoti Mazumder

**Affiliations:** 1 Department of Mechanical Engineering, College of Engineering, University of Michigan, Ann Arbor, 48109-2136 USA; 2 Department of Materials Science and Engineering, College of Engineering, University of Michigan, Ann Arbor, 48109-2136 USA

## Abstract

Bimodal ultrafine eutectic composites (BUECs) exhibit a good combination of strength and plasticity owing to a dual-hierarchy in eutectic length-scales in the microstructure. The present study investigates the variation of phase, morphology, feature length-scales and modality of microstructures obtained in a Al_81_Cu_13_Si_6_ (at. %) ternary alloy after laser surface remelting. A novel approach of varying component bimodal eutectic volume fractions by controlling the cooling rate of the laser solidification process has been presented. The volume fraction of the fine eutectic matrix has a profound effect on the flow strength. Laser remelted microstructures with volume fractions of the fine eutectic varying from 25 to 40% exhibiting compressive flow strengths ranging from 500 to 900 MPa have been obtained. The volume fraction of the fine eutectic decreased with cooling rate and completely ceased to exist at cooling rates greater than $4\times {10}^{4}\,^\circ {\rm{C}}/{\rm{s}}$.

## Introduction

Most ultrafine and nanoscale grained metallic alloys exhibit high strength but show poor plastic deformability^1^. There is a trade-off between strength and ductility associated with materials processing techniques. Bimodal ultrafine eutectic composites (BUECs), characterized by a heterogeneous microstructure with a dual hierarchy in length-scales, coarse sub-micron dendritic colonies dispersed in a nanoncrystalline eutectic matrix, have been reported to exhibit a good combination of strength and plasticity. These structures were first developed in Ti-based alloys^2^. BUECs exhibit a good combination of strength, strain hardening and plasticity in several ternary and quaternary alloy systems^2–16^. The high strength results from dislocation confinement within the ultrafine eutectic matrix^17,18^. The good compressive plastic behavior originates from abundant slip/shear bands in the coarse dendrites^12^, obstruction of runaway shear bands at the dendrite-matrix interface^19^ and rotation of ultrafine matrix along primary shear band direction^6^. The formation of BUECs is favored under high cooling rates; methods like rolling at low temperature^20^, arc melting^14,15^, induction melting^16^, suction casting into a copper mold^2–9^ and semi-solid sintering^12^ have been used to fabricate BUECs in the past. The present study employed rapid solidification via laser surface remelting to study the effects of cooling rate on the microstructure and properties of Al_81_Cu_13_Si_6_ ternary alloy.

Any solidification process with a cooling rate greater than 10^2^ K/s can be considered as rapid solidification^21^. Rapid solidification leads to remarkable non-equilibrium effects within the fusion zone such as grain refinement, extended solid solubility and evolution of metastable crystalline phases^22^. Rapidly solidified materials exhibit improvements in mechanical properties such as strength^23–26^, fracture toughness^27,28^ and super plasticity^29–32^. These materials also exhibit enhanced corrosion resistance^33–35^ and respond better to subsequent working or heat treatment processing^36^. Laser materials processing is associated with high cooling rates, as high as 10^10^ K/s with ultrashort pulsed laser beams^37^. This occurs due to rapidly moving temperature fields and results in the departure from local equilibrium conditions at the solid/liquid interface^38^. The principles of full diffusional (equilibrium) solidification cannot be directly applied because of the introduction of morphological instabilities and formation of metastable phases^39^.

Laser surface remelting is a versatile rapid solidification technique which can be employed to realize a wide range of processing conditions (cooling rates) by varying parameters such as laser power and scan velocity. Refined microstructures with an interlamellar spacing of up to 17 nm have been obtained in Al-Cu binary eutectic system^40^. Using solidification modelling techniques, the morphology of Al-Cu-Si system under rapid solidification conditions has been studied only in alloys with low Si content (1–2 at. %)^39^. Experimental investigations on the Al-rich part of Al-Cu-Si system using casting methods have resulted in bimodal type structures^5–7,41^. The emphasis of the previous works has been on mechanical properties and deformation mechanisms of as-cast structures fabricated with minimal process control^5,7,8^. There is at present little information about processing conditions conducive for bimodal eutectic structure formation.

In the present work, we report the effect of processing conditions on the phase, morphology, modality, volume fraction and feature length-scales of solidification microstructure of Al-Cu-Si BUECs. In the past, the primary method for the control of volume fraction involved varying the alloy composition^4,8,14^. However, the processing conditions and mechanical properties of Al_81_Cu_13_Si_6_ BUECs with varying volume fractions have not been studied till date. We leveraged the versatile cooling rate capabilities of laser surface remelting to vary critical BUEC microstructure attributes within the melted zone. The hardness and compressive flow strength of the microstructures was investigated. The possible solidification paths under non-equilibrium conditions were discussed to explain the variation in microstructure phase, morphology and volume fraction. This work also presents the upper bound of cooling rate range favourable for BUEC formation; the fine nanocrystalline matrix ceased to form beyond this limit.

## Results

### Microstructure of as-received samples

**Figure 1:**
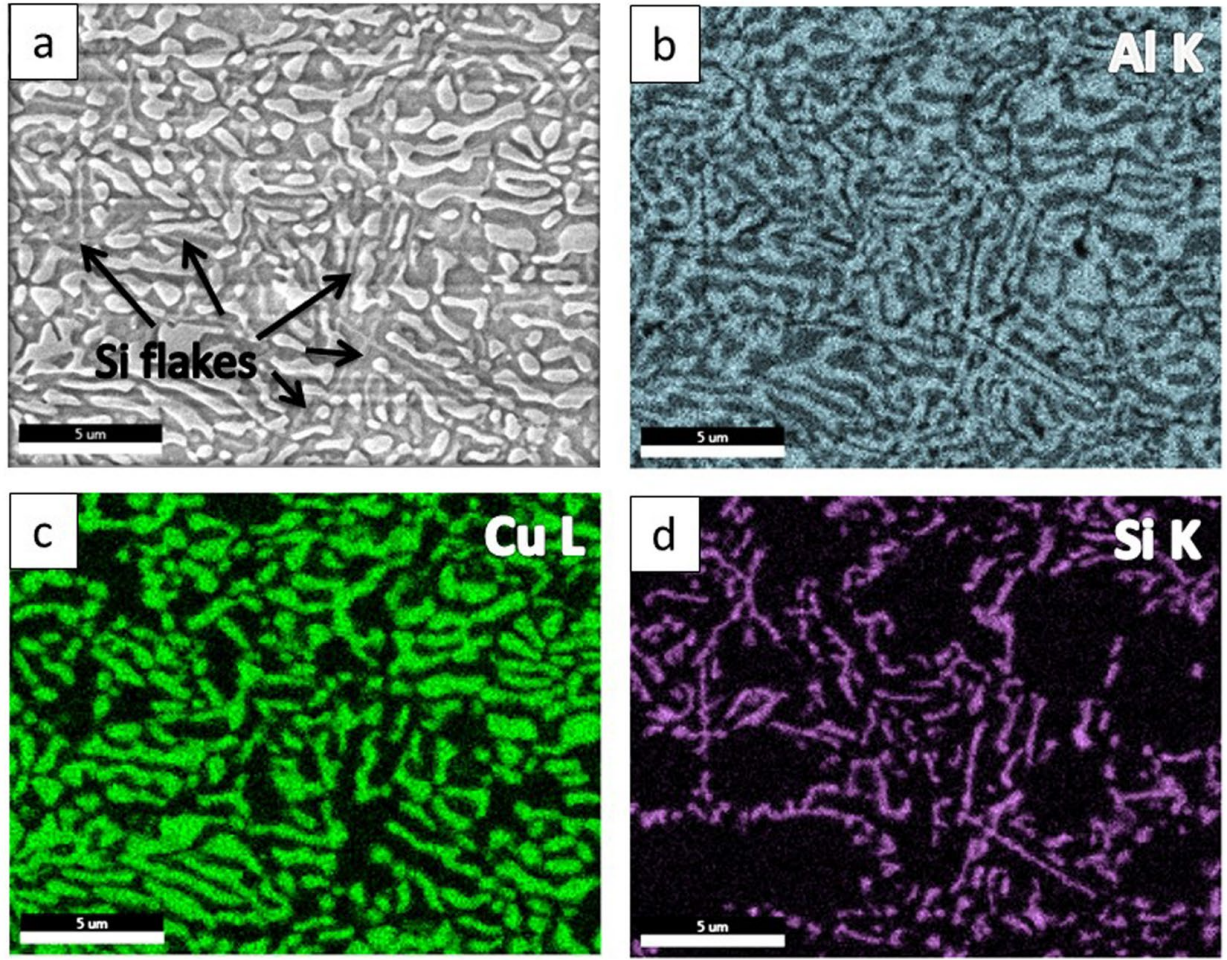
(**a**) SEM image of as-cast ternary alloy; EDX mapping of different elements: (**b**) Al; (**c**) Cu; (**d**) Si.

The substrate for laser surface remelting was arc cast with a composition of Al_81_Cu_13_Si_6_. Figure 1 shows the SEM micrograph of the as received material and the EDX mapping of the primary elements, Al, Cu and Si. The SEM micrograph of the substrate revealed a ternary eutectic structure with θ – Al_2_Cu and Si phases dispersed uniformly in α – Al matrix. The length scales of the θ – Al_2_Cu phase ranged from a few 100 nanometers to a few 10 microns, at different regions in the material.

### Microstructure of laser treated samples

**Figure 2:**
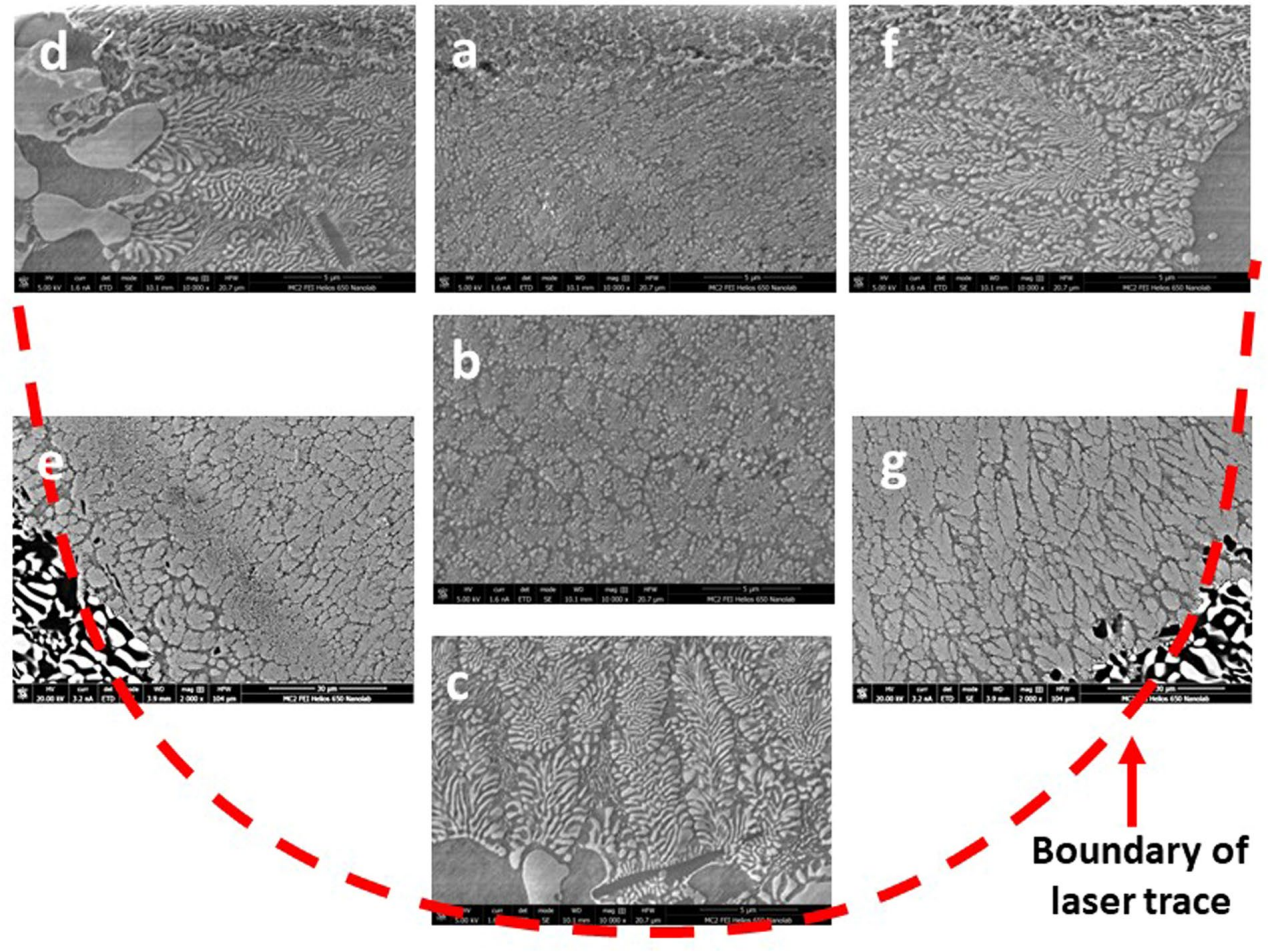
SEM images of microstructures: (**a–c**) along laser trace centreline and (**d–f**) along laser trace boundary. Process parameters of sample A: P = 1000 W, S_s_ = 0.8 mm, S_v_ = 25.4 mm/s.

**Figure 3:**
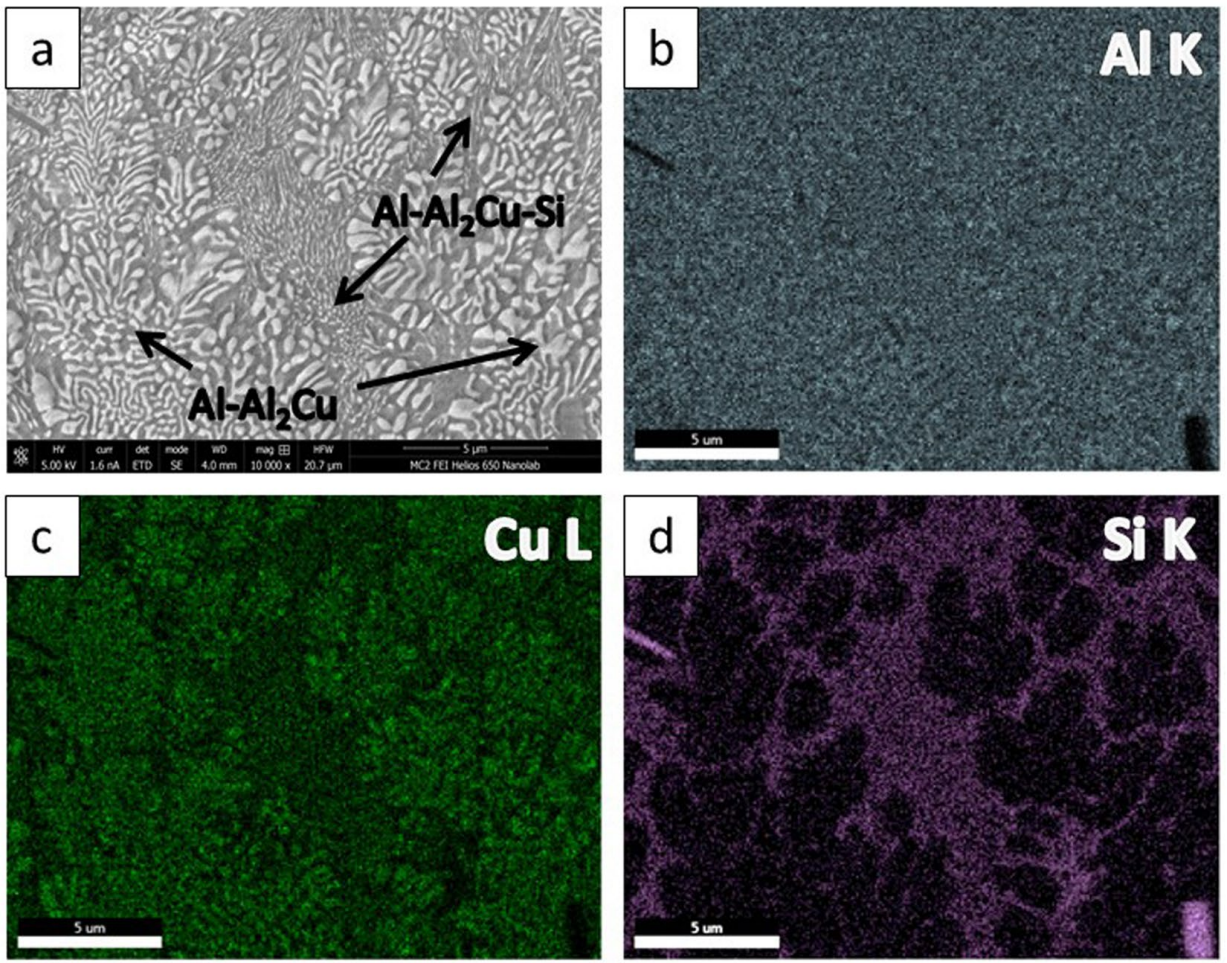
Microstructure of the bottom region of sample A: (**a**) SEM image of bimodal eutectic (coarse phase: Al-Al_2_Cu and fine matrix: Al-Al_2_Cu-Si), EDX element mapping: (**b**) Al; (**c**) Cu; (**d**) Si.

**Figure 4:**
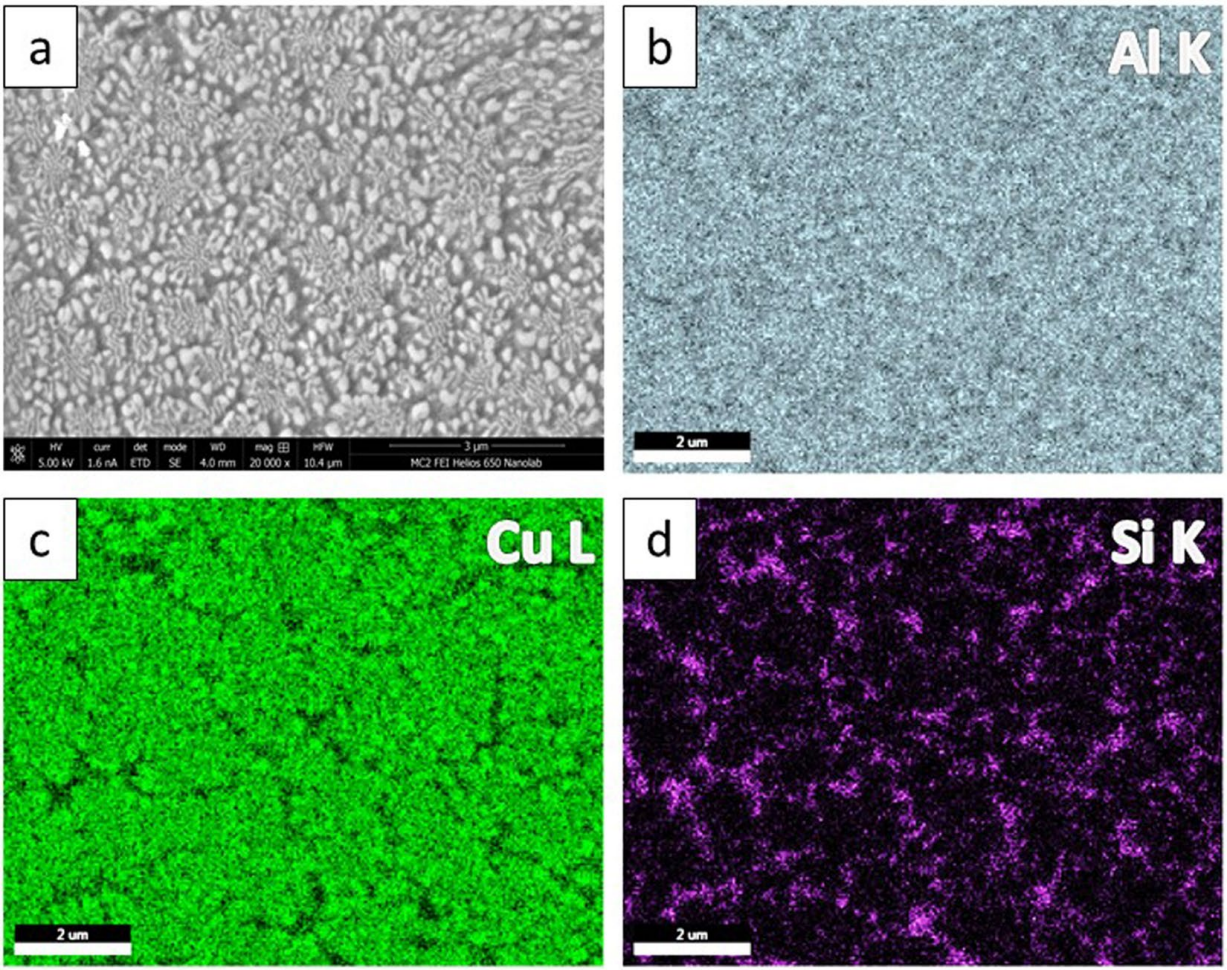
Microstructure of the top region of sample A: (**a**) SEM image of dual-binary eutectic (coarse phase: Al-Al_2_Cu and fine matrix: Al-Si), EDX element mapping: (**b**) Al; (**c**) Cu; (**d**) Si.

**Figure 5:**
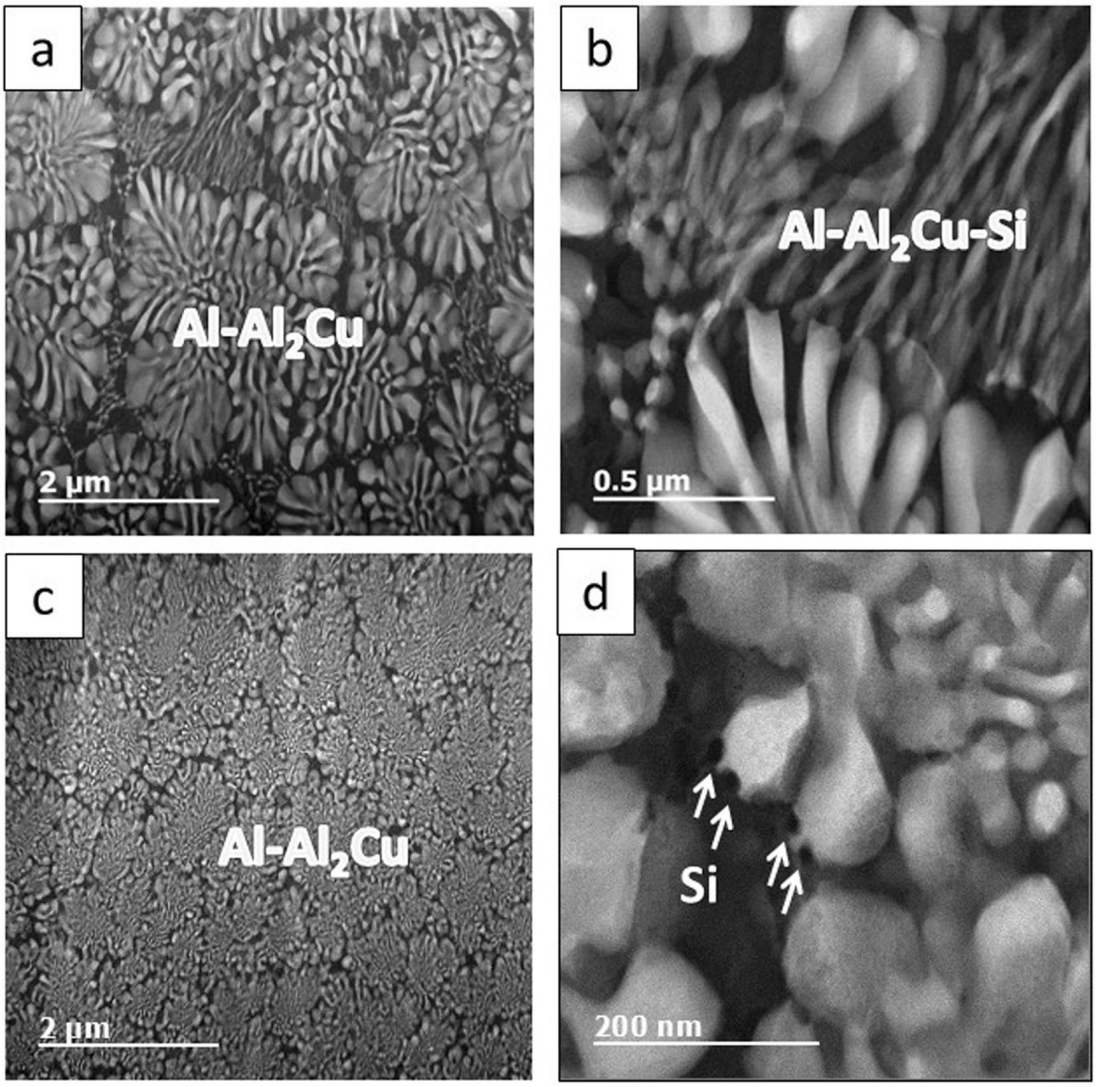
HAADF TEM images of two types of eutectic structure, (**a**,**b**) Bimodal eutectic: coarse phase: Al-Al_2_Cu and fine matrix: Al-Al_2_Cu-Si, (**c,d**) Dual-binary: coarse phase: Al-Al_2_Cu and fine matrix: Al-Si. (images from sample A).

The phase, morphology, length scale and modality of the solidification microstructure varied significantly at different locations within the laser trace. Figure 2 shows the SEM micrographs of the microstructures obtained at various regions within the melt pool, i.e. along the boundary **(**Fig. 2d–g) and center-line of the laser trace **(**Fig. 2a–c). The corresponding laser process parameters were power (P): 1000 W, spot size (S_S_): 0.8 mm and scanning velocity (S_V_): 25.4 mm/s (sample A). A bimodal eutectic structure with two distinct phases with different length-scales of lamellar spacing was observed near the boundary. The coarse eutectic (binary) consisted of α – Al and θ – Al_2_Cu phases while the fine eutectic (ternary) consisted of α – Al, θ – Al_2_Cu and Si phases. Figure 3 shows the SEM micrograph and EDX mapping of the Al, Cu and Si in the bimodal eutectic structure (sample A). Si was primarily confined to the fine matrix. The coarse eutectic had a discontinuous morphology with interphase spacings in the 100–400 nm range; the coarsest structures were obtained along the trace boundary. The volume fraction of the fine eutectic was highest along the trace boundary and gradually decreased towards the interior regions of the laser trace. In the interior regions of the laser trace, extending to the top, the microstructure of previously fine ternary eutectic structure transformed to a binary structure of Si dispersed in an α – Al matrix. The bimodal nature of eutectic length-scales ceased to appear in this region. Thus, this microstructure can be considered as a ‘dual-binary eutectic’. Figure 4 shows the SEM micrograph and EDX mapping of the microstructure without an interdendritic ternary eutectic phase. The volume fraction of the binary Al – Si structure was about 5–10%, significantly lower than the volume fraction of the ternary structure observed at the melt pool boundary (40–45%). Figure 5 shows the high-angle annular dark-field (HAADF) TEM images of the two microstructure types. In the binary/ternary bimodal eutectic **(**Fig. 5a,b **)**, the lamellar spacing of Al-Al_2_Cu eutectic was larger as compared to the dual-binary eutectic **(**Fig. 5c,d **)**. The absence of the θ – Al_2_Cu phase in the Si rich regions indicated that Cu was restricted to the coarse eutectic phase **(**Fig. 5d **)**. The finest Al-Al_2_Cu eutectic structure was obtained near the top of the laser trace, on the laser beam axis.

### Hardness and flow strength

**Figure 6:**
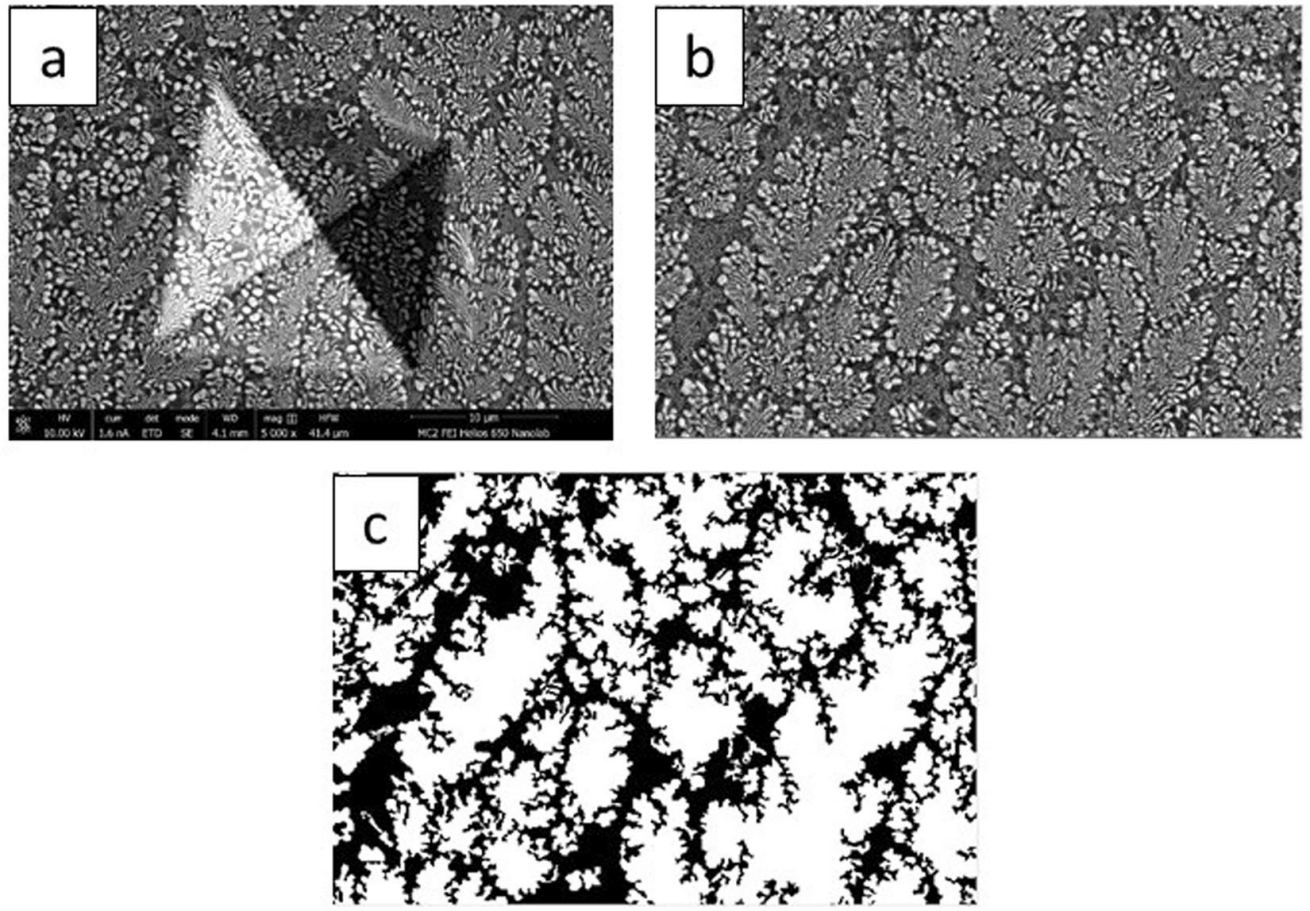
(**a**) SEM image of indent location comprising of both coarse and fine eutectic, (**b**) SEM image of area near the indent location, used for volume fraction measurement, (**c**) Processed SEM image (dark areas can be mapped to fine eutectic region). (images from sample A).

**Figure 7:**
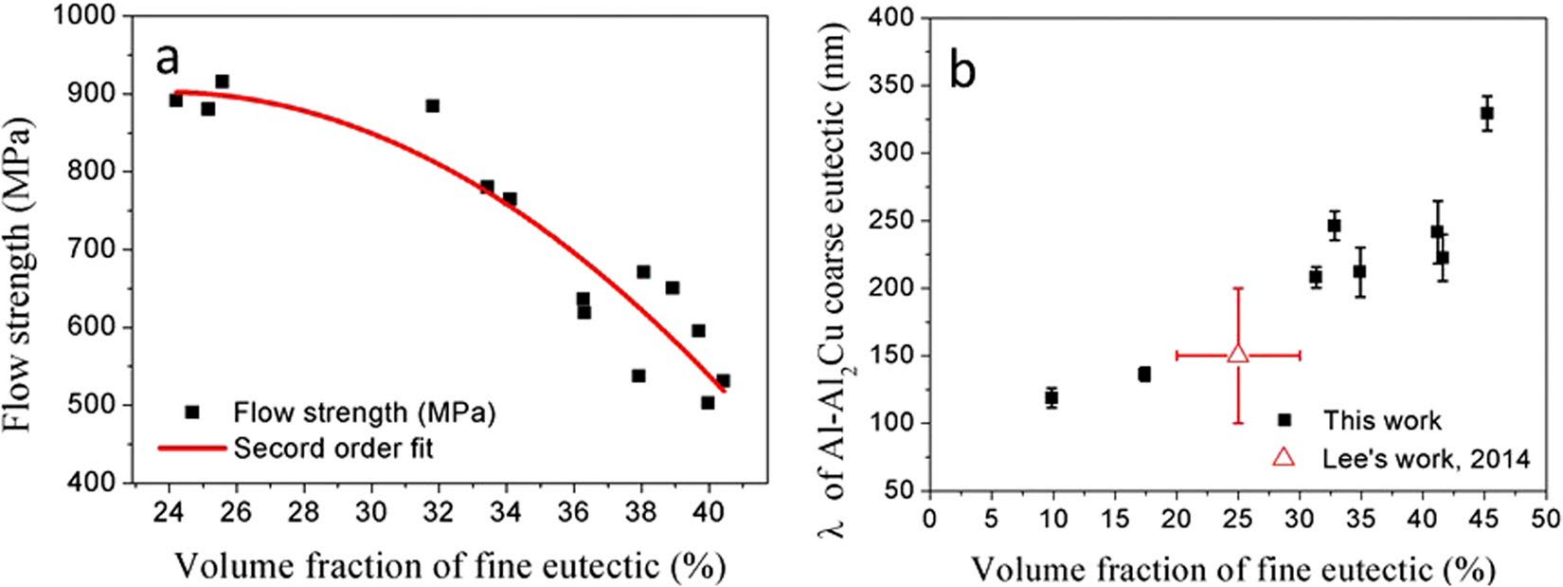
(**a**) Variation of flow strength with volume fraction of the fine eutectic in bimodal eutectic type microstructure (coarse eutectic: Al + Al_2_Cu, fine matrix: Al + Al_2_Cu + Si); (**b**) Variation interlamellar spacing of Al-Al_2_Cu coarse eutectic with volume fraction of fine eutectic matrix. Process parameters: P = 500–1000 W, S_s_ = 0.8–1.2 mm, S_v_ = 2.54–101.4 mm/s.

The Vickers hardness was measured at several locations within the laser trace and as-received sample. The variation of measured hardness (and flow strength estimated from hardness) with volume fraction of the fine eutectic was also studied. Figure 6 shows the methodology adopted to study the effect of volume fraction on the flow strength of the bimodal microstructure (sample A). The projected indent area was measured at each indent location. Furthermore, an SEM image adjacent to the indent was captured to estimate the volume fraction of the fine eutectic structure. The varying contrast between the coarse and fine eutectics was leveraged to estimate volume fractions at multiple locations (with an accuracy of $\pm$5%). The SEM micrograph at each location was transformed to a binary image using image processing functions in *MATLAB* ^TM^ and the volume fraction of the black pixels (fine eutectic region) was obtained. The flow strength was estimated as indentation hardness (in GPa) divided by a factor of 2.7^42^. The laser process parameters were varied to obtain microstructures with different volume fractions of the fine eutectic (see *Materials and methods* section). Figure 7a shows the variation of flow strength with volume fraction of the fine eutectic. The flow strength varied from 500 to 900 MPa for fine eutectic volume fractions ranging from 25 to 40%. Figure 7b shows the variation of interlamellar spacing in the coarse eutectic with volume fraction of the fine matrix. The Al-Al_2_Cu eutectic became coarser at higher volume fractions of the fine eutectic. The flow strength of the ternary (as-received) and binary eutectic regions were approximately 609 ± 5  MPa and 1033 ± 10  MPa respectively; finer feature length-scales resulted in higher flow strengths in these regions.

## Discussion

The cooling rate at different locations within the melt pool can be drastically different^43,44^. This resulted in a non-uniform solidification microstructure, in terms of phase, morphology and feature length scales. The highest cooling rates were realized on the centerline of the fusion zone while the lower cooling rates were obtained along the boundary. Thus, fine grained microstructures were obtained at the top of the laser trace along the beam axis and the radial coarsening of grains was observed away from this region^45^. The modification of grain size was also accompanied by a change in the eutectic phase and morphology, a direct result of non-equilibrium solidification at high cooling rates (>10^2^ °C/s).

### Solidification paths for ternary alloy

The solidification of ternary alloys is complex, with several phases and morphologies possible based on the alloy composition and growth conditions^46^. The formation of phases with specific morphologies under non-equilibrium conditions can be treated by evaluating competitive growth through interface response functions (interface temperature). Upon satisfying thermodynamic stability criteria, the phase and morphology with the highest interface temperature grows^47^. Three types of eutectic structures attain stability during laser surface melting of Al_81_Cu_13_Si_6_ ternary alloy and they each grow under a different range of overlapping processing conditions. They are Al-Al_2_Cu-Si ternary eutectic, Al-Al_2_Cu binary eutectic and Al-Si binary eutectic.

In the ternary phase diagram (liquidus projection) of Al-Cu-Si, the binary eutectic point appears as univariant equilibrium lines which meet at ternary invariant point. In the present work, the composition of Al_81_Cu_13_Si_6_ coincides with a ternary invariant point which signifies that the four phases (Al, Al_2_Cu, Si and liquid) are in equilibrium at the eutectic temperature of 524 °C. The substrate was arc-cast at relatively lower cooling rates compared to laser melting and can be considered to be closer to the liquidus projection phase diagram. The solidification microstructure of the as-received sample was certainly Al-Al_2_Cu-Si ternary eutectic which was confirmed from the SEM micrograph.

Rapid solidification is a non-equilibrium phenomenon which may result in the undercooling of melt, formation of metastable phases, and coring or microsegregation in the solid solution^21^. The solidification microstructure within the laser trace was therefore expected to deviate significantly from the equilibrium ternary phase diagram prediction. However, at lower scan velocities (<5 mm/s), a fine Al-Al_2_Cu-Si ternary eutectic was obtained within the laser trace.

The coupled zone refers to the range of compositions and growth conditions (non-equilibrium) where the solidification microstructure is wholly eutectic^48^. Within the coupled zone, the eutectic structures grow faster than the competing dendritic phases. This concept is primarily applicable to binary systems but can be extended to ternary systems as well^41^. There exists an invariant coupled zone within which the formation of a ternary eutectic phase is more feasible than competing dendritic structures (Al–Al_2_Cu in this case). Outside the invariant coupled zone, the formation of Al–Al_2_Cu dendrites is observed. A univariant coupled zone can be associated with this binary eutectic reaction. The morphology of the Al–Al_2_Cu eutectic is not lamellar since the solidification process is associated with high growth velocities in the presence of a third component (Si). The destabilization of the planar growth interface under rapid solidification conditions can be treated by an absolute stability condition for undercooled alloy melts, which considers the surface energy effect in addition to the constitutional undercooling criterion^49^. Morphological instability is further induced by the formation of a long range solute boundary layer, resulting from the segregation of the third component (Si)^50^.

At higher cooling rates, the solidification path proceeds along the univariant equilibrium line associated with the following binary eutectic reaction:

1 $$L\to \alpha ({\rm{Al}})+{\rm{\theta }}({{\rm{Al}}}_{2}{\rm{Cu}})$$

Al and Al_2_Cu dendrites with primary arm spacings ranging from 100–500 nm coprecipitate as a binary eutectic mixture. When the interdendritic melt composition reaches the invariant ternary eutectic composition at 524 °C, it is quenched, resulting in the formation of a fine nanocrystalline matrix^41^.

2 $$L\to \alpha ({\rm{Al}})+{\rm{\theta }}({{\rm{Al}}}_{2}{\rm{Cu}})+{\rm{Si}}$$

The final solidification microstructure closely resembled those reported by earlier studies on Al-Cu-Si bimodal eutectic composites^6–8^. However, in this study, the feature length scales and volume fractions of constituent eutectics varied significantly within the laser trace. The previous investigations report a small subset of the structures obtained in this study owing to limitations in their solidification processing capabilities. The dendritic arm spacing of the coarse binary eutectic and the volume fraction of the fine matrix were dependent on the solidification conditions (cooling rate). The volume fraction of the fine matrix decreased with finer length scales of the coarse binary eutectic. A shift in primary phase boundary of the coupled zone at high growth velocities has been observed in binary systems^51^. Similarly, the coupled zone associated with the ternary eutectic reaction shifts as the growth velocity increases. The growth velocity increases with distance from the trace bottom, explaining the observed microstructure modality and phase distribution. The evolution of a ternary fine eutectic matrix was favored at lower growth velocities.

### Effect of cooling rate on solidification microstructure

**Figure 8:**
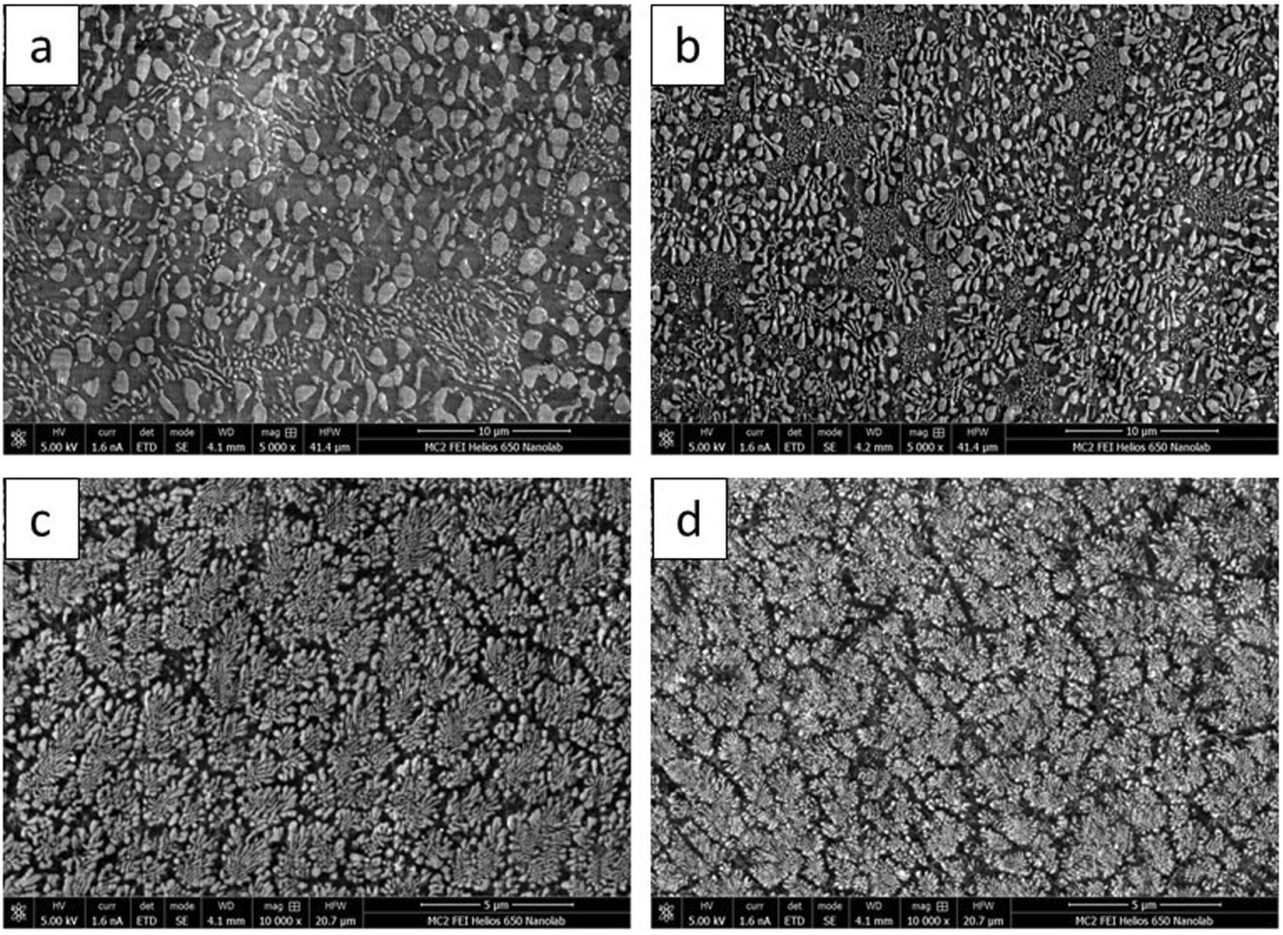
Microstructures obtained at the top of laser trace corresponding to cooling rates of (**a**) $3.8\times {10}^{3}\,^\circ {\rm{C}}/{\rm{s}}$; (**b**) $14\times {10}^{3}\,^\circ {\rm{C}}/{\rm{s}}$; (**c**) $49\times {10}^{3}\,^\circ {\rm{C}}/{\rm{s}}$; (**d**) $54\times {10}^{3}\,^\circ {\rm{C}}/{\rm{s}}$. Process parameters: P = 1000 W. S_s_ = 0.8 mm. S_v_ = (**a**) 2.54 mm/s; (**b**) 5.28 mm/s; (**c**) 20.32 mm/s; (**d**) 25.4 mm/s.

The cooling rate of the solidification process had a significant effect on the morphology and phase distribution of the solidification microstructure. However, this change in morphology and elemental distribution was restricted to the phase containing Si, i.e. the ternary Al-Cu-Si and binary Al-Si phases. The cooling rate of the solidification process was experimentally obtained to study its effect on the microstructure phase, morphology and modality. The average cooling rate and associated microstructures (captured by SEM) are shown in Fig. 8. The samples were laser treated by a laser power of 500 W, spot size of 0.8 mm, and different scanning speeds of 2 54, 5.28, 20.32, and 25.4 mm/s. The ternary eutectic structure ceased to form at a cooling rate of $\sim 4\times {10}^{4}\,^\circ {\rm{C}}/{\rm{s}}$. The bimodal nature of the microstructure was not observed at higher cooling rates; with Si globules precipitating along the Al − Al_2_Cu eutectic colony interfaces. This cooling rate also signifies the limit of the invariant coupled zone of the ternary eutectic solidification process. The decrease in spacing of Al − Al_2_Cu eutectic colonies was continuous regardless of the existence of the fine ternary eutectic matrix. The consistent dendritic morphology of the Al − Al_2_Cu eutectic colonies and absence of any primary phase indicates that the solidification conditions were still confined to the univariant coupled zone associated with the Al − Al_2_Cu binary eutectic reaction.

### Tuning flow strength of BUECs

The flow strength of the solidified microstructure depends on the feature length scales via a Hall-Petch type relationship. Bimodal eutectic structures with lower volume fractions of the fine eutectic exhibited higher flow strengths. The higher flow strengths are a result of the reduced spacings in both the coarse and fine eutectic structures, obtained under solidification at high cooling rates. Thus, the spacings and volume fractions of both eutectics in BUECs must be considered in understand the overall flow strength. A strict dependence between the coarse eutectic spacing and fine eutectic volume fraction was observed over a wide range of solidification conditions, realized by varying laser scanning speed and laser power. This result revealed that any attempt to refine the microstructure would result in reduction of volume fraction of fine eutectic. The heterogeneity of length scales in the solidification microstructure reduced and eventually disappeared as the cooling rate was increased. The bimodal nature suppresses shear localization and crack nucleation, improving compressive plastic strain^5^. The shear stress across interfaces between the coarse and fine eutectic colonies is effectively dissipated by the rotation of the coarse eutectic colonies^6^. These effects are likely to be less pronounced at lower volume fractions of the fine eutectic. The high compressive plastic strain which results from this heterogeneity of length scales is expected to be compromised in structures solidified at high cooling rates. Thus, the strength and compressive plasticity can be tuned over a wide range by varying the cooling rate of the solidification process.

The findings of this study will enable more precise control of mechanical properties of rapid solidification processing (e.g. laser surface melting, suction casting, metal based additive manufacturing etc.) of eutectic ternary alloys. The large variation in flow strength and possibly ductility (not addressed in this work) over a narrow range of cooling rates could result in BUECs finding application in materials processing environments with dynamic requirements. Further studies are required to establish the effects of varying temperature gradients and growth velocities on the morphology and length-scales of BUECs. These parameters could offer more precise control of microstructure attributes such as feature length-scales and volume fraction; thus, providing a robust processing technique for BUECs with a wide range of mechanical properties.

## Summary and Conclusions

The variation of morphology, constituent phases and length-scales of an arc-melted Al_81_Cu_13_Si_6_ alloy upon laser remelting have been studied. Three distinct eutectic structures were obtained based on location within the laser trace: Al-Al_2_Cu-Si ternary eutectic, Al-Al_2_Cu binary eutectic and Al-Si binary eutectic. The extent of departure from equilibrium solidification (quantified by cooling rate) determined the solidification microstructure, which was either a (i) ternary eutectic, or (ii) binary (Al-Al_2_Cu)/ternary (Al-Al_2_Cu-Si) bimodal eutectic, or (iii) ‘dual-binary (Al-Al_2_Cu)/Al-Si eutectic’. The formation of bimodal eutectics was favored at cooling rates under $\sim 4\times {10}^{4}\,^\circ {\rm{C}}/{\rm{s}}$. The flow strength of bimodal eutectics was effectively varied between 500 to 900 MPa, a result of differing fine eutectic volume fractions within the laser trace. The control of volume fraction and constituent phases will provide greater flexibility in processing materials with diverse mechanical properties.

## Materials and Methods

### Sample preparation and laser surface remelting

A eutectic Al_81_Cu_13_Si_6_ (at. %) ternary alloy ingot was fabricated using an arc melter (Materials Preparation Center, Ames Laboratory, Iowa State University). Argon gas environment was employed to protect the sample from oxidation during arc melting. Specimens with a thickness of 20mm × 10mm × 2 mm were cut from the as-cast ingot.

After grinding and polishing, the specimens were laser surface remelted using solid state disk laser (TRUMPF Laser HLD 4002) at a wavelength of 1030 nm. Previous investigations on laser surface remelting of Al alloys primarily used CO_2_ laser (λ = 10.6 μm)^40,52^. However, the absorption coefficient of Al at this wavelength is low (2–3%)^53^. Lower wavelength laser irradiation resulted in a more efficient remelting process (absorption coefficient of 10%), permitting the use of lower powers and higher scan speeds. The absorption was further improved by coating the sample with graphite (Bonderite L-GP G aerosolized graphite lubricant) prior to remelting. The laser power (P) was varied between 500 and 1000 W. The laser spot diameter (S_s_) and scan speed (S_y_) ranged from 0.8 to 1.2 mm and 2.54 to 101.4 mm/s respectively. Argon shielding gas (flow rate of 9.4 L/min) was used during the laser melting process to prevent oxidation. A two-color pyrometer (IMPAC ISR 12-LO/GS) was used to measure the temperature-time history at the top of the melt pool. The temperature history was obtained at approximately the middle of laser scan path, to avoid anomalous free-edge heat transfer effects.

### Materials characterization and hardness testing

The eutectic microstructures of the as-cast and laser treated Al-Cu-Si specimens were characterized using an FEI Helios 650 scanning electron microscope (SEM) in the secondary electron mode. The TEM samples were prepared by mechanical cutting, grinding, polishing followed by focussed ion beam milling. High angle annular dark field (HAADF) images and corresponding bright field images were captured using a JEOL 3100 R05 TEM with an operating voltage of 300 kV. Indentation experiments were conducted at room temperature on a hardness tester. A Vickers indenter (Clark CM-800 microhardness tester) was employed to perform the indentation experiments in the load controlled mode, with the peak load ranging from 250 mN to 2 N.

### Image processing

The coarse and fine eutectic phases were mapped to white and black pixels respectively using imadjust and *medfilt2* MATLAB functions. The image was further processed to fill gaps using erosion and dilation operations with a disk structuring element. The *imcomplement* and *imfill* functions were used to fill any remaining holes. The volume fraction of the fine eutectic phase was evaluated as the number of black pixels divided by the total number of pixels.

## References

[1] Gleiter H (2000). Nanostructured materials: basic concepts and microstructure. Acta Mater..

[2] He G, Eckert J, Löser W, Schultz L (2003). Novel Ti-base nanostructure–dendrite composite with enhanced plasticity. Nat. Mater..

[3] Misra D, Rakshit R, Singh M, Shukla P (2014). High yield strength bulk Ti based bimodal ultrafine eutectic composites with enhanced plasticity. Mater.Des..

[4] Das J (2007). Bulk ultra-fine eutectic structure in Ti-Fe-base alloys. J. Alloys Compd..

[5] Park JM (2009). High-strength bulk Al-based bimodal ultrafine eutectic composite with enhanced plasticity. J. Mater. Res..

[6] Lee SW (2015). Micro-to-nano-scale deformation mechanisms of a bimodal ultrafine eutectic composite. Sci. Rep..

[7] Park JM (2010). Multi-phase Al-based ultrafine composite with multi-scale microstructure. Intermetallics.

[8] Kim JT (2016). Understanding the relationship between microstructure and mechanical properties of Al-Cu-Si ultrafine eutectic composites. Mater. Des..

[9] Han JH (2008). Influence of a bimodal eutectic structure on the plasticity of a (Ti 70.5 Fe29.5)91 Sn9 ultrafine composite. Appl. Phys. Lett..

[10] Park JM (2008). High strength Ni-Zr binary ultrafine eutectic-dendrite composite with large plastic deformability. Appl. Phys. Lett..

[11] Park JM (2008). High strength ultrafine eutectic Fe-Nb-Al composites with enhanced plasticity. Intermetallics.

[12] Yang C (2017). Bimodal titanium alloys with ultrafine lamellar eutectic structure fabricated by semi-solid sintering. Acta Mater..

[13] Cao GH (2014). Formation of a bimodal structure in ultrafine Ti-Fe-Nb alloys with high-strength and enhanced ductility. Mater. Sci. Eng. A.

[14] Han JH (2011). Effect of microstructure modulation on mechanical properties of Ti-Fe-Sn ultrafine eutectic composites. Met. Mater. Int..

[15] Sun BB (2006). Ultrafine composite microstructure in a bulk Ti alloy for high strength, strain hardening and tensile ductility. Acta Mater..

[16] Song GA (2009). Mechanical properties of large-scale Mg-Cu-Zn ultrafine eutectic composites. J. Alloys Compd..

[17] Nieh T, Wadsworth J (1991). Hall-Petch relation in nanocrystalline solids. Scr. Metall. Mater..

[18] Pande C, Masumura R, Armstrong R (1993). Pile-up based Hall-Petch relation for nanoscale materials. Nanostructured Mater..

[19] Ma E (2003). Controlling plastic instability. Nat. Mater..

[20] Wang Y, Chen M, Zhou F, Ma E (2002). High tensile ductility in a nanostructured metal.Nature..

[21] Otooni, M. *Elements of rapid solidification: fundamentals and application*s. (2013).

[22] Liebermann, H. Rapidly solidified alloys: processes, structures, properties, applications. Marcel Dekker, Inc, 270 Madison Ave, New York, New (1993).

[23] Kawamura Y, Hayashi K, Inoue A (2001). Rapidly solidified powder metallurgy Mg97Zn1Y2alloys with excellent tensile yield strength above 600 MPa. Metal Trans..

[24] Correia J, Davies H, Sellars C (1997). Strengthening in rapidly solidified age hardened Cu-Cr and Cu-Cr-Zr alloys. Acta Mater..

[25] Sanctis MD (1991). Structure and properties of rapidly solidified ultrahigh strength Al-Zn-Mg-Cu alloys produced by spray deposition. Mater. Sci. Eng. A..

[26] Maeng D, Kim T, Lee J, Hong S, Seo S (2000). Microstructure and strength of rapidly solidified and extruded Mg-Zn alloys. Scr. Mater..

[27] Hooreweder BV, Moens D, Boonen R (2012). Analysis of fracture toughness and crack propagation of Ti6Al4V produced by selective laser melting. Advanced..

[28] Zhang H, He Y, Pan Y (2013). Enhanced hardness and fracture toughness of the laser-solidified FeCoNiCrCuTiMoAlSiB 0.5 high-entropy alloy by martensite strengthening. Scr. Mater..

[29] Mabuchi M, Kubota K, Higashi K (1995). High strength and high strain rate superplasticity in a Mg-Mg 2 Si composite. Scr. Metall. Mater..

[30] Cui Z, Zhong W, Wei Q (1994). Superplastic behavior at high strain rate of rapidly solidified powder metallurgy Al-Li alloy. Scr. Metall. Mater..

[31] Fujino S, Kuroishi N, Yoshino M, Mukai T, Okanda Y (1997). High-strain-rate superplastic behavior in a super-rapidly-solidified Al-Si system alloy. Scr. Mater..

[32] Ruano O, Eiselstein L, Sherby O (1982). Superplasticity in rapidly solidified white cast irons. Metall. Trans. A..

[33] Yamasaki M, Hayashi N, Izumi S, Kawamura Y (2007). Corrosion behavior of rapidly solidified Mg-Zn-rare earth element alloys in NaCl solution. Corros. Sci..

[34] Makar G, Kruger J (1990). Corrosion studies of rapidly solidified magnesium alloys. J. Electrochem. Soc..

[35] Izumi S, Yamasaki M, Kawamura Y (2009). Relation between corrosion behavior and microstructure of Mg-Zn-Y alloys prepared by rapid solidification at various cooling rates. Corros. Sci..

[36] Nussbaum G, Sainfort P, Regazzoni G, Gjestland H (1989). Strengthening mechanisms in the rapidly solidified AZ 91 magnesium alloy. Scr. Metall..

[37] Allmen MV, Huber E, Blatter A, Affolter K (1984). Melt Quenching at 10 exp 10 at K/s. Int. J. Rapid Solidif..

[38] Kurz, W. & Fisher, D. Fundamentals of solidification, *Trans Tech Publ. Switz*. (1986)

[39] Gilgien P, Zryd A, Kurz W (1995). Metastable Phase Diagrams and Rapid Solidification. ISIJ Int..

[40] Gill SC, Zimmermann M, Kurz W (1992). Laser resolidification of the AlAl2Cu eutectic: The coupled zone. Acta Metall. Mater..

[41] de Wilde J, Froyen L (2006). Microstructures observed during directional solidification along the univariant eutectic reaction in a ternary Al-Cu-Si alloy. Solidif. Gravity Iv.

[42] Mata M, Anglada M, Alcalá J (2002). Contact deformation regimes around sharp indentations and the concept of the characteristic strain. J. Mater. Res..

[43] Chande T, Mazumder J (1984). Estimating effects of processing conditions and variable properties upon pool shape, cooling rates, and absorption coefficient in laser welding. J. Appl. Phys..

[44] Raghavan A, Wei HL, Palmer TA, DebRoy T (2013). Heat transfer and fluid flow in additive manufacturing. J. Laser Appl..

[45] Kou, S. Welding metallurgy. *New York* (1987).

[46] McCartney D, Hunt J, Jordan R (1980). The structures expected in a simple ternary eutectic system: Part 1. Theory. Metall. Mater..

[47] Kurz W, Gilgien P (1994). Selection of microstructures in rapid solidification processing. Mater. Sci. Eng. A.

[48] Kurz W, Fisher D (1979). Dendrite growth in eutectic alloys: the coupled zone. Int. Met. Rev..

[49] Trivedi R, Kurz W (1986). Morphological stability of a planar interface under rapid solidification conditions. Acta Metall..

[50] De Wilde J, Froyen L, Rex S (2004). Coupled two-phase [α(Al) + θ(Al2Cu)] planar growth and destabilisation along the univariant eutectic reaction in Al-Cu-Ag alloys. Scr. Mater.

[51] Pierantoni M, Gremaud M, Magnin P, Stoll D (1992). The coupled zone of rapidly solidified Al-Si alloys in laser treatment. Acta Metall..

[52] Gill S, Kurz W (1993). Rapidly solidified Al-Cu alloys-I. experimental determination of the microstructure selection map. Acta Metall. Mater..

[53] Larcombe, D. Fibre versus CO_2_ laser cutting. *AILU e-newsletter* (2014).

